# Rational design and characterization of enhanced alcohol-inducible synthetic promoters in *Pichia pastoris*

**DOI:** 10.1128/aem.02191-24

**Published:** 2024-12-19

**Authors:** Qi Liu, Yun-hao Li, Liu-fei Tao, Jia-yi Yang, Yi-lun Zhang, Meng-hao Cai

**Affiliations:** 1 State Key Laboratory of Bioreactor Engineering, East China University of Science and Technology 47860 , Shanghai, China; 2 Shanghai Collaborative Innovation Center for Biomanufacturing, Shanghai, China; Chalmers tekniska hogskola AB, Gothenburg, Sweden

**Keywords:** *Pichia pastoris*, methanol, ethanol, synthetic promoter

## Abstract

The C1 and C2 alcohols hold great promise as substrates for biomanufacturing due to their low cost and rich resources. *Pichia pastoris* is considered a preferred host for methanol and ethanol bioconversion due to its natural utilization of methanol and ethanol. However, the scarcity of strong and tightly regulated alcohol-inducible promoters limits its extended use. This study aimed to develop enhanced methanol- and ethanol-inducible promoters capable of improving gene expression in *P. pastoris*. Rational design strategies were employed to rewire the upstream regulatory sequence of the methanol-inducible P*_AOX1_*, generating several high-strength methanol-inducible promoters with a stringent regulatory pattern. Eleven strong promoters were identified from 36 endogenous ethanol-inducible candidates recognized from transcriptome analysis. Core promoter regions, the crucial element influencing transcriptional strength, were also characterized. Five high-activity core promoters were then combined with four upstream regulatory sequences of high-strength promoters, resulting in four groups of synthetic promoters. Ultimately, the highly active methanol-inducible P*_A13_* and ethanol-inducible P*_0688_* and P*_synIV-5_* were selected for the expression of an α-amylase and yielded enzyme activity 1.6, 2.6, and 4.5 times higher as compared to that of P*_AOX1_*. This work expands the genetic toolkit available for *P. pastoris*, providing more precise and efficient options for regulating gene expression. It benefits the use of *P. pastoris* as an efficient platform for the C1 and C2 alcohol-based biotransformation in industrial biotechnology.

**IMPORTANCE:**

*P. pastoris* represents a preferred microbial host for the bio-utilization of C1 and C2 alcohols that are regarded as renewable carbon sources based on clean energy. However, lack of efficient and regulated expression tools highly limits the C1 and C2 alcohols based bioproduction. By exploring high-strength and strictly regulated alcohol-inducible promoters, this study expands the expression toolkit for *P. pastoris* on C1 and C2 alcohols. The newly developed methanol-inducible P*_A13_* and ethanol-inducible P*_synIV-5_* demonstrate significantly higher expression levels than the commercial P*_AOX1_* system. The endogenous and synthetic promoter series established in this study provides new construction references and alternative tools for expression control in *P. pastoris* for C1 and C2 alcohols based biomanufacturing.

## INTRODUCTION

C1 and C2 alcohols, i.e., methanol and ethanol, have notable advantages as substrates for biomanufacturing and bioconversion, owing to their low cost, wide availability, and high energy density (1–4). Both C1 and C2 alcohols can be synthesized from CO_2_, making them promising candidates for next-generation clean energy and substrates (3, 5, 6). Compared to traditional substrates like glucose, methanol has a higher reduction degree, which is beneficial for product synthesis (1, 7, 8). Ethanol, on the other hand, is easily metabolized into acetyl-CoA, providing sufficient precursor for microbial growth and product synthesis (2, 9–11). These benefits have driven researchers to develop efficient bioconversion systems utilizing methanol and ethanol.

*Pichia pastoris* (syn. *Komagataella phaffii*) is considered an ideal chassis for methanol biotransformation due to its natural methanol metabolic pathways and powerful methanol assimilation capacity (1, 4, 7, 12). Furthermore, *P. pastoris* exhibits efficient ethanol metabolism, enabling the synthesis of various products from ethanol (2, 9–11). Numerous studies have demonstrated that *P. pastoris* is a versatile chassis host for methanol and ethanol biotransformation. For efficient biotransformation, enzyme expression control by strong and tightly regulated promoters is often required (13). While *P. pastoris* possesses several effective methanol-inducible promoters (14), ethanol-inducible promoters are quite limited (9, 15). Moreover, the scarcity of strong and tightly regulated promoters limits further improvements in biotransformation efficiency of *P. pastoris* (13, 16).

To present, different transcriptional regulatory tools for C1 and C2 alcohols biotransformation have been developed in *P. pastoris* (9–11, 17–21). However, most high-strength transcriptional tools require modifications to cellular regulatory networks or the design of complex genetic circuits (9, 19–24). In complicated regulation cases particularly the pathway control, simpler and more straightforward transcriptional tools are preferred. Therefore, many studies focused on the modification and reconstruction of high-strength and tightly regulated promoters, especially the methanol-inducible *alcohol oxidase 1* promoter (P*_AOX1_*) (21, 25–29). Deletions, insertions, and mutations in sequences have been performed to obtain higher-strength P*_AOX1_* variants. As transcription factors and their binding sites on the P*_AOX1_* have been identified (30–32), the rational promoter modifications become predictable and feasible. In contrast, research on ethanol-inducible promoters in *P. pastoris* still remains limited. This study rationally modifies and reconstructs the upstream regulatory sequence (URS) of P*_AOX1_* based on the distribution of transcription factor-binding sites to select higher-strength methanol-inducible promoter mutants. On the other hand, transcriptome analysis was used to identify strong and tightly inducible ethanol promoters. High-activity endogenous core promoters were further screened and combined with high-activity URSs to construct synthetic alcohol-inducible promoters. With these efforts, we aim to offer flexible and efficient expression tools for C1 and C2 alcohols based biomanufacturing in *P. pastoris*.

## RESULTS

### Redesign of upstream regulatory sequence of P*_AOX1_*

**Fig 1:**
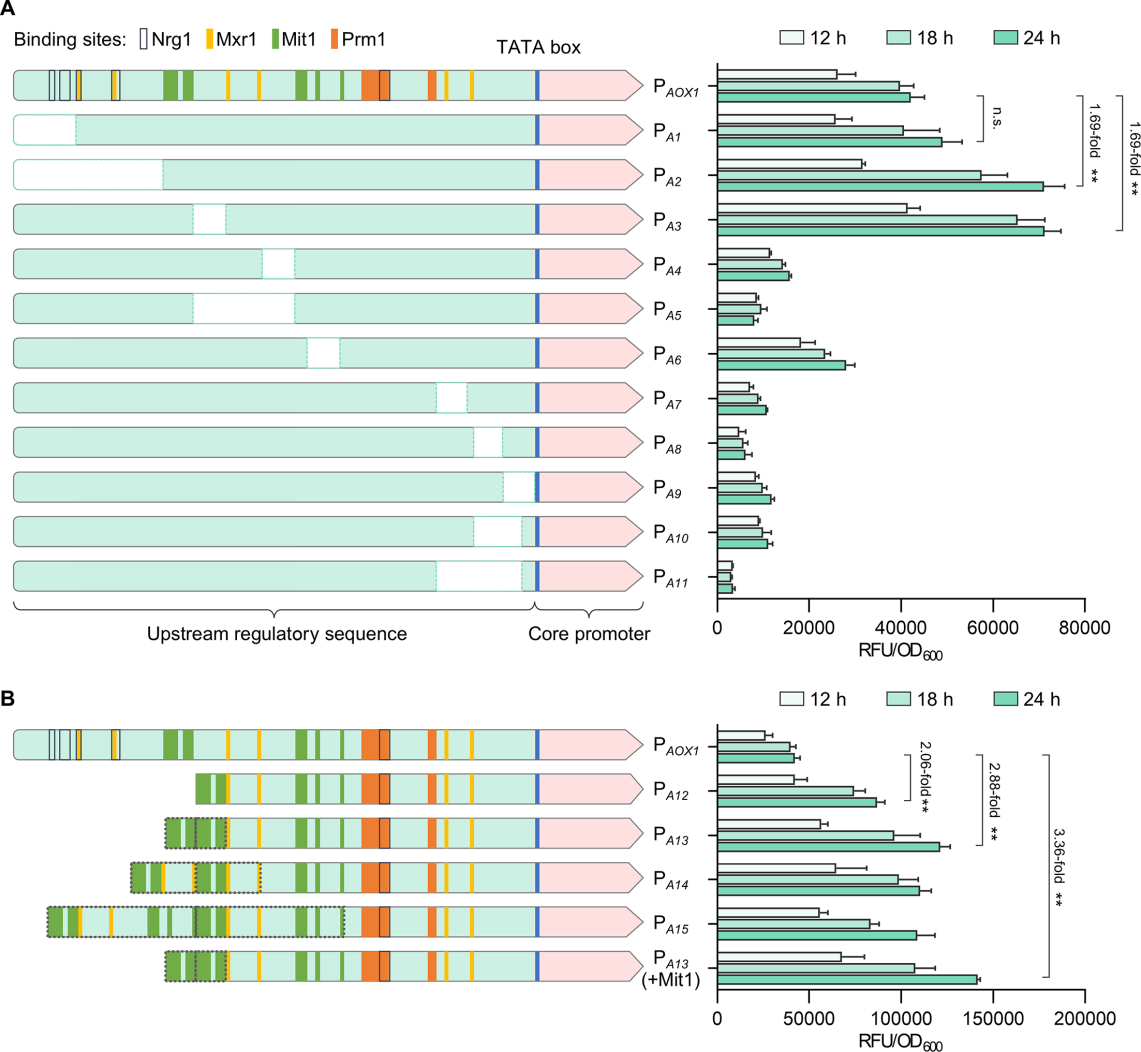
Redesign and characterization of URS of P*_AOX1_*. (**A**) The URS of the P*_AOX1_* promoter was systematically truncated, resulting in a series of deletion mutants (P*_A1_* to P*_A11_*). The binding sites for key transcription factors (Nrg1, Mxr1, Mit1, Prm1, details in Fig. S1) and the TATA box are highlighted in different colors. (**B**) The mutant P*_A12_* was constructed by combining the deletion sequences of two high-strength mutants, P*_A2_* and P*_A3_*. Additional 5′ end duplications of P*_A12_* generated three more mutants (P*_A13_*, P*_A14_*, and P*_A15_*). The duplication sequences are marked with dashed boxes. Mit1 was overexpressed by P*_GAP_* to further improve the activity of P*_A13_*. Then, the GFP intensity of the strains was measured after cultured in YNM medium. Statistical significance of GFP intensity of each strain at 24 h is shown (**P < 0.01).

The P*_AOX1_* is the most widely used methanol-inducible promoter in *P. pastoris*. Previous studies have identified binding sites of transcription factors within the URS of P*_AOX1_*, allowing rational reconstruction of the URS. Our previous study proved that activators of Mxr1, Prm1, and Mit1 had multiple binding sites within the URS, while the repressor Nrg1 showed two independent binding sites and three overlapping binding sites with Mxr1 and Prm1, respectively (Fig. 1A; Fig. S1). These results clarified the basic mechanism that supporting the rational rewiring of the URS.

To enhance the activity of the promoter, we initially deleted the region containing the Nrg1-binding sites at the 5′ end of P*_AOX1_*, generating two mutants: P*_A1_* (Δ−940~−848 bp) and P*_A2_* (Δ−940~−718 bp) (Fig. 1A). The green fluorescent protein (GFP) was used as a reporter to evaluate promoter activity. Fluorescence detection showed that the deletion of two independent Nrg1-binding sites did not increase P*_A1_* activity significantly; but, the deletion of four Nrg1 binding sites significantly increased P*_A2_* activity by 68.7% despite the simultaneous deletion of two Mxr1-binding sites. We then segmented and deleted unknown functional sequences between the transcriptional activator binding sequences in the URS, producing nine mutants from P*_A3_* to P*_A11_* (Fig. 1A). Considering that the deletion of Mxr1 binding sites did not negatively affect the mutant P*_A2_*, we deleted the long regions containing Mxr1 binding sites in P*_A5_* and P*_A9_*. All the deletions led to significant activity reduction but not the mutant P*_A3_*. The mutant P*_A3_* (Δ−672~−624 bp) showed a 69.1% increase in activity relative to the P*_AOX1_*. Next, we combined the most effective deletions from the high-activity mutants P*_A2_* and P*_A3_* to create the mutant P*_A12_*, which exhibited 2.06-fold activity as compared to the P*_AOX1_* (Fig. 1B). To further enhance promoter strength, we separately duplicated the specific segments of 45 bp (containing two Mit1 binding sites), 97 bp (containing two Mit1 and two Mxr1 binding sites), and 222 bp (containing five Mit1 and two Mxr1 binding sites) from the 5′ end of P*_A12_*, resulting in mutants P*_A13_*, P*_A14_*, and P*_A15_* (Fig. 1B). The results showed that P*_A13_* exhibited 2.88-fold activity relative to the P*_AOX1_*, but no further increase in strength was observed from P*_A14_* (2.62-fold) and P*_A15_* (2.58-fold) despite that additional duplication of Mit1 and Mxr1 binding sequences were added. As more Mit1 binding sites were added, they may recruit more Mit1 for this rewired promoter. On the other hand, our group and other groups reported that Mit1 overexpression obviously enhanced P*_AOX1_* activity (19, 24, 33). Therefore, we then overexpressed Mit1 to further improve the activity of P*_A13_*. This strategy increased the intensity of P*_A13_* by 16% (Fig. 1B), achieving 3.36-fold activity relative to the P*_AOX1_*.

Subsequently, seven high-strength mutant promoters were evaluated for activity under different carbon sources to assess regulatory stringency. Despite some Nrg1 binding site were deleted, all the seven improved mutants maintained low basal expression under glucose, glycerol, and ethanol conditions (Fig. S2). In summary, through rational design and reconstruction, we generated a P*_AOX1_* mutant toolbox and identified URS mutants with enhanced activity and high regulatory stringency, which can be applied in subsequent experimental designs.

### Screening of ethanol-inducible promoters in *P. pastoris*

**Fig 2:**
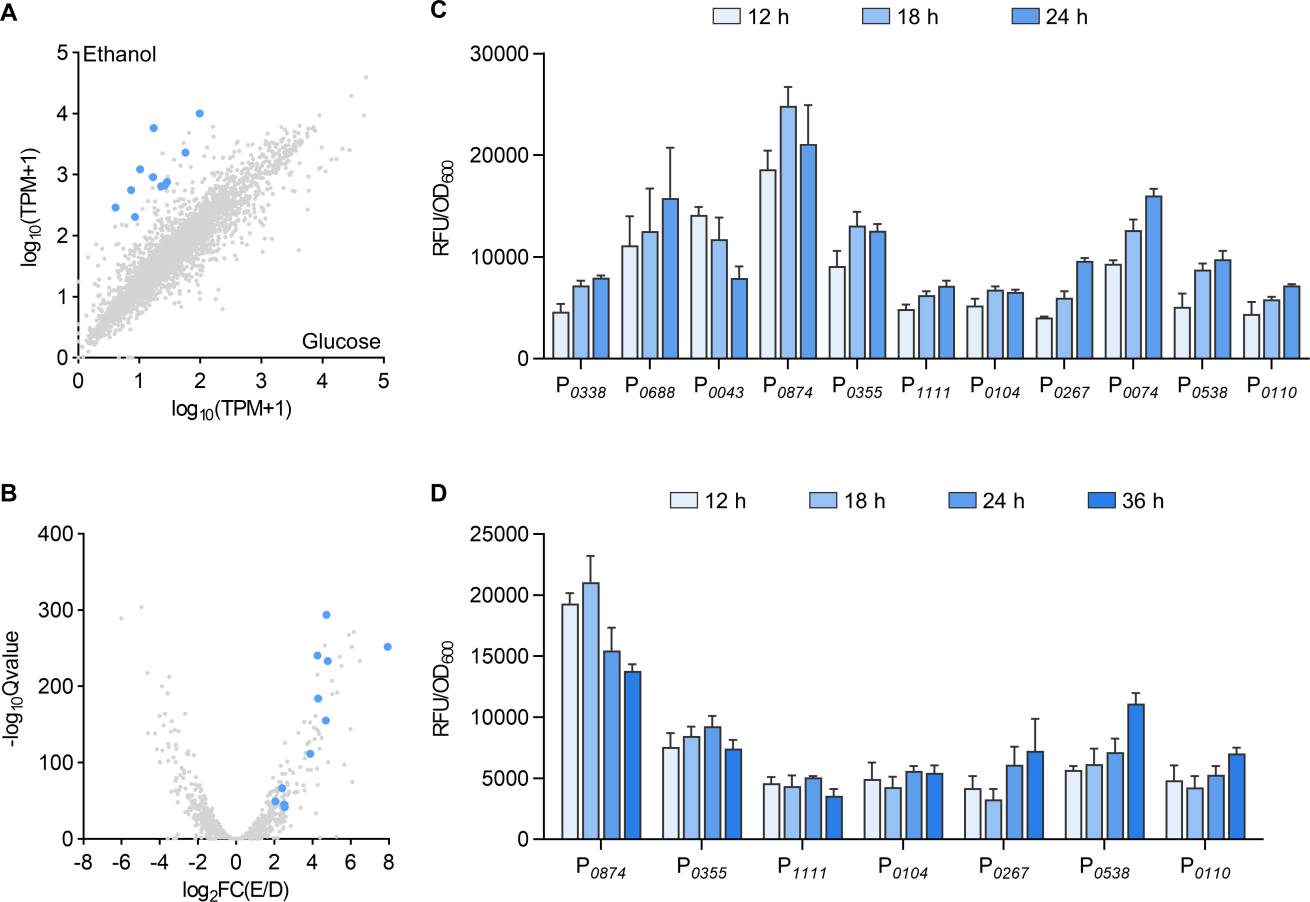
Screening and characterization of ethanol-inducible promoters. The scatter plot (**A**) and volcano plot (**B**) of transcriptomic comparison of *P. pastoris* wild-type strain GS115 grown in glucose and ethanol conditions. Eleven promoters with good expression levels were selected from a total of 36 endogenous promoters screened by RNA-Seq analysis (Fig. S3) and marked with blue dots in (**A**) and (**B**). (**C**) The GFP intensity of the 11 selected promoters was measured after cultured in YNE medium. (**D**) The GFP intensity of the seven promoters responsive to both ethanol and methanol was measured after cultured in the YNEM medium.

Unlike the abundance of methanol-inducible promoters, ethanol-inducible transcription tools are relatively scarce in *P. pastoris*. In order to identify natural promoters responsive to ethanol, we performed transcriptome sequencing on the wild-type strain GS115 under glucose and ethanol conditions. Differential gene expression analysis indicated genes with significantly higher expression in ethanol in comparison to glucose (ethanol/glucose_foldchange >5, TPM_ethanol >500) (Fig. 2A and B). A 1,000 bp sequence upstream of the start codon of these genes was chosen as the promoter region to drive GFP expression. A total of 36 endogenous ethanol-inducible promoters were identified, and their activities were tested by GFP under ethanol conditions (Fig. S3; Table S1). Among them, 11 high-strength promoters were ultimately selected for further activity assessment under different carbon sources (Fig. 2C; Fig. S4). Consistent with the transcriptome data, the P*_0874_* showed the highest activity under ethanol conditions but also with remarkable leakage expression under glucose condition. In contrast, the P*_0688_* and P*_0074_* demonstrated high activity under ethanol conditions with low leakage, making them more suitable for ethanol-inducible gene expression. Additionally, some promoters also demonstrated high expression levels under methanol condition, with the P*_0104_* and P*_0110_* exhibiting even higher activity in methanol than in ethanol (Fig. S4). We selected seven promoters with high expression levels under both methanol and ethanol conditions. Their activities were assessed by a mixed methanol-ethanol feeding strategy, revealing that promoter activity under mixed methanol-ethanol substrates closely matched that under ethanol conditions (Fig. 2D). These findings provide valuable insights and references for their industrial applicability.

### Construction and characterization of synthetic promoters

**Fig 3:**
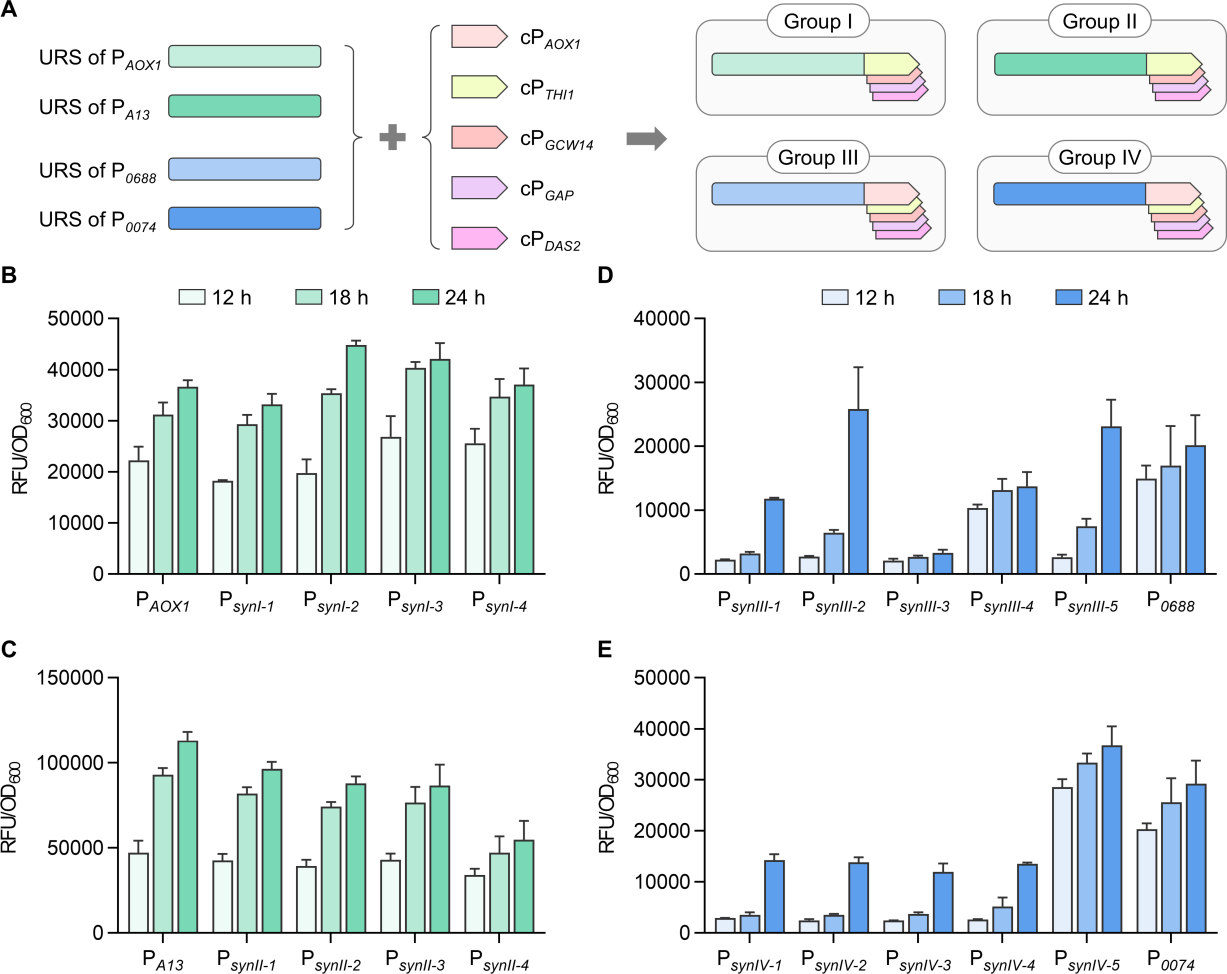
Design and characterization of synthetic promoters with high activity URSs and core promoters. (**A**) A schematic diagram illustrating the combination of four highly active URSs with five highly active core promoters, resulting in four groups of synthetic promoters. The activities of synthetic promoters in group I (**B**) and group II (**C**) were measured after cultured in YNM medium. The activities of synthetic promoters of group III (**D**) and group IV (**E**) were measured in after cultured YNE medium.

In addition to URS, core promoter regions also play a crucial role in determining promoter strength. While the *AOX1* core promoter (cP*_AOX1_*) is frequently studied and modified, other native core promoters have been comparatively less explored. Therefore, 12 native strong promoters (13, 14, 34–39) were selected, with the region from 20 bp upstream of the TATA box to the start codon site defined as the core promoter region (Fig. S5A). These core promoters were combined with the URS of P*_AOX1_* to generate synthetic promoters, with their activity measured under methanol conditions by GFP fluorescence detection (Fig. S5B). Four core promoters (cP*_THI11_*, cP*_GCW14_*, cP*_GAP_*, cP*_DAS2_*) showed higher activity than the cP*_AOX1_*. These four, along with cP*_AOX1_*, were combined with the URS of P*_AOX1_* and three previously identified high-activity promoters (P*_A13_*, P*_0688_*, and P*_0074_*), resulting in four groups of synthetic promoters (Fig. 3A). Group I and Group II were induced with methanol, using P*_AOX1_* and P*_A13_* as controls, respectively. Group III and Group IV were induced with ethanol, using P*_0688_* and P*_0074_* as controls, respectively. In Group I, all synthetic promoters displayed activities comparable to P*_AOX1_* (Fig. 3B). In contrast, in Group II, the synthetic promoters showed lower activity than the P*_A13_*, suggesting that URS modifications can impact the compatibility between URS and core promoters (Fig. 3C). Notably, two synthetic promoters in Group III showed higher activity than the P*_0688_*, but their induction was delayed, with significant fluorescence intensity observed only 24 h (Fig. 3D). In Group IV, synthetic promoters generally had lower activity, with only the P*_synIV-5_* reaching 1.2 times as that of the P*_0074_* (Fig. 3E). Although Group I and Group II had similar URS, synthetic promoter strength trends were inconsistent, suggesting URS and core promoter functions are interdependent. Additionally, the leaky expression of all synthetic promoters under glucose condition was evaluated, showing that these promoters maintained strict regulation, with regulatory patterns closely associated with their upstream regulatory sequences (Fig. S6).

### Expression of α-amylase by synthetic alcohol-inducible promoters

**Fig 4:**
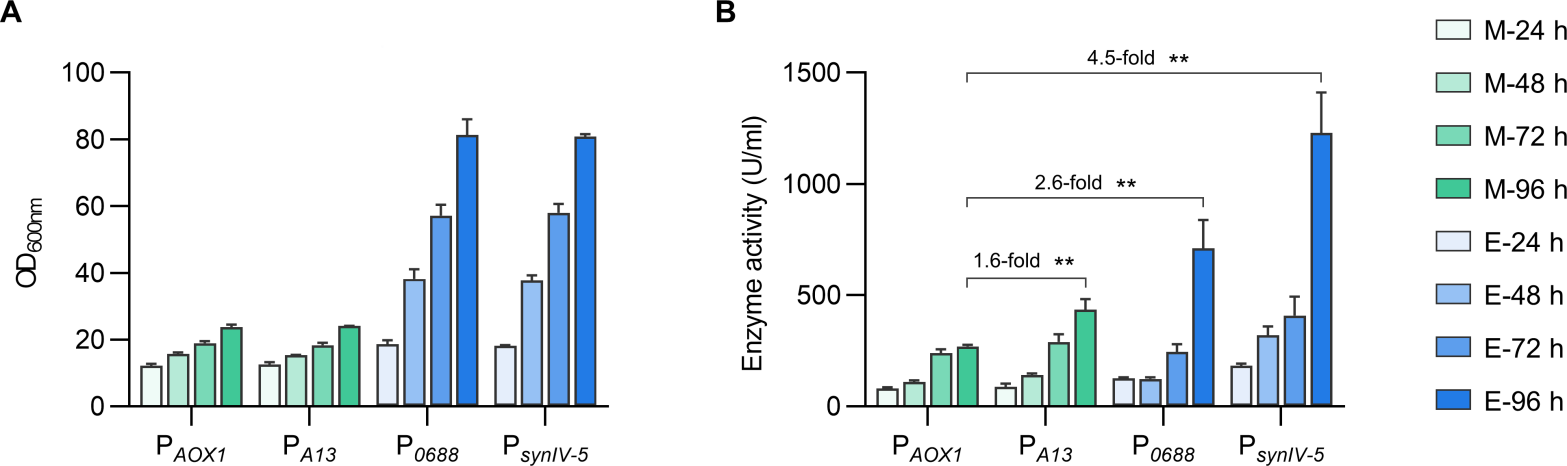
Evaluation of high-activity promoters on α-amylase production. Methanol-inducible P*_AOX1_* and P*_A13_*, along with ethanol-inducible P*_0688_* and P*_IV-5_*, were employed to express α-amylase. The OD_600nm_ of culture broth (**A**) and enzyme activity of α-amylase (**B**) were measured every 24 h. Statistical significance of enzyme activity of each strain at 96 h is shown (**P < 0.01).

Considering promoter strength and response sensitivity, we selected methanol-inducible P*_A13_* and ethanol-inducible P*_0688_* and P*_synIV-5_* to express an α-amylase of deep-sea origin (40), with the same protein expressed under P*_AOX1_* as the control, to evaluate their performance in protein expression. Both strains showed similar growth trends under the same carbon source conditions, suggesting that the synthetic promoters did not impose an additional burden on cellular regulatory networks (Fig. 4A). Enzyme activity driven by P*_A13_* under methanol was 1.6 times higher than that driven by P*_AOX1_*. Under ethanol condition, enzyme activity driven by the P*_0688_* and P*_synIV-5_* were 2.6 and 4.5 times higher than that driven by P*_AOX1_*, respectively (Fig. 4B). These results differed from fluorescence assay, likely due to substantial cell growth under ethanol condition. Overall, these engineered methanol- and ethanol-inducible promoters showed better expression capacity in comparison to the widely used P*_AOX1_* in *P. pastoris*.

## DISCUSSION

The C1 and C2 alcohols, which can be produced in large quantities through catalytic conversion of syngas components (such as CO and CO_2_) or from various renewable resources, have the potential to serve as renewable feedstocks for the future biomanufacturing industry (1, 2, 5, 7). *P. pastoris*, with its efficient methanol and ethanol metabolism, serves as an ideal chassis for alcohol biotransformation. Developing effective induction tools that decouple cell growth from product synthesis is crucial for optimizing microbial cell factories. In this study, we expanded the alcohol-inducible expression toolkit in *P. pastoris* by screening and engineering a range of native and synthetic promoters with varying strengths. These efficient tools can well support *P. pastoris* as a superior chassis platform for green biomanufacturing and bioconversion.

The reconstruction of the P*_AOX1_* has consistently attracted significant attention. In this study, using a rational design strategy targeting the URS of P*_AOX1_*, we successfully generated several mutants with enhanced promoter strength while maintaining stringent regulation. The URS of P*_AOX1_* has been a longstanding research focus, but prior random deletion strategies hardly provided significant activity improvements (27, 29). In contrast, our approach, combining the deletion of repressor binding sites and the duplication of activator binding sites, led to a 2.88-fold increase in comparison to the P*_AOX1_* activity, highlighting the significance of the −717 to −673 region. The promoter remodeling strategies in this study could provide valuable reference and assistance for further research on P*_AOX1_* and artificial modification of other promoters (41, 42). Despite deleting four repressor sites, the mutated promoters maintained stringent regulation, presenting them as strong methanol-inducible tools, potentially replacing the commercial P*_AOX1_* system. However, the deletion of non-transcription factor binding sequences in the −672 to −163 region had no positive effect, confirming that these sequences, though not bound by transcription factors, play significant roles in P*_AOX1_* activity. The base mutation strategy (25, 28) could be applied in this region, alongside the deletions from this study, to potentially achieve a more potent methanol-inducible promoter in future.

Beyond promoter modification, some studies have explored transcription factor reprogramming to improve expression efficiency and regulatory flexibility in *P. pastoris* (19, 21, 33, 43). For example, overexpression of activators, knock-out of repressors, and dynamic control of transcription factor expression. In this study, Mit1 overexpression further enhanced the activity of P*_A13_*, illustrating the compatibility of promoter engineering with transcription factor reprogramming. Therefore, the combined use of *cis*-acting element engineering and *trans*-acting element rewiring can improve promoter activity synergistically, which will certainly facilitate the development of customizable and high-performance promoters.

Ethanol-driven biosynthesis in *P. pastoris* has been extensively studied, showing promising applications (9–11). However, the scarcity of robust ethanol-responsive regulatory tools remains a major bottleneck in developing ethanol-based biosynthesis systems. We addressed this by conducting transcriptomic analyses to identify ethanol-inducible promoters with diverse expression strengths and regulatory stringency, offering valuable tools for ethanol-based biotransformation. Further screening and core promoter replacement significantly enhanced the activity of these ethanol-inducible promoters. Notably, the P*_synIV-5_* under ethanol outperformed the P*_AOX1_* under methanol in protein expression, making it a promising new tool for ethanol-driven production. Additionally, we identified and characterized dual carbon source-inducible promoters (e.g., P*_0104_* and P*_0110_*) with comparable expression under ethanol and methanol conditions, offering greater flexibility and broader application potential for *P. pastoris* in alcohol utilization and conversion.

The interdependence between the URS and core promoter regions also played a crucial role in determining promoter strength. While the URS is essential for regulating the transcriptional response to external signals, the core promoter governs the recruitment of basal transcriptional machinery, directly affecting overall promoter strength (44, 45). Our study revealed that the compatibility between URS and core promoters is not always straightforward. For example, synthetic promoters combining high-activity URS with various core promoters exhibited inconsistent performance, with some constructs even displaying lower activity than expected (Fig. 3C). These findings suggest that URS modifications may affect core promoter functionality in ways that are not fully predictable, possibly due to changes in chromatin structure or transcription factor dynamics. Therefore, future promoter engineering efforts should consider the complex interplay between URS and core promoter regions to achieve optimal expression.

## MATERIALS AND METHODS

### Plasmids, strains, and growth conditions

The plasmids pP-P*_AOX1_*G and pPlacO1cAG were previously constructed in our laboratory (23). Other plasmids used in this study were constructed by Gibson assembly. Details of plasmid construction can be found in the supplemental material. All plasmids were linearized with *Sal*I and transformed into competent cells of *P. pastoris* wild-type strain GS115. The plasmids and strains used in this study are listed in Tables S2 and S3. Primers used for the construction of plasmids were synthesized by Beijing Tsingke Biotech Co., Ltd., China, and listed in Table S4. *Escherichia coli* was cultured at 37°C in LB medium (0.5% yeast extract, 1% tryptone, and 1% NaCl). 100 µg/mL ampicillin was added when required. *P. pastoris* was cultured at 30°C in YPD (1% yeast extract, 2% tryptone, and 2% glucose) medium for cell growth, or YND (0.67% YNB, 0.5% glucose) plate for transformant screening.

### Transcriptome analysis

The wild-type *P. pastoris* GS115 was pre-cultured in YPD medium to a log phase and then separately shifted to YNE medium (0.67% YNB, 0.5% ethanol) and YND medium supplement with histidine of 50 µg/mL. Each group was cultured independently in triplicate. Cells were collected at 6 h for RNA extraction and transcriptome sequencing, which was performed by Majorbio (Shanghai, China). High-quality reads were aligned onto the indexed GS115 reference genome (GCF_000027005.1_ASM2700v1) (46). Bioinformatic analysis was performed using the online tools of Majorbio Cloud Platform (http://www.majorbio.com).

### GFP fluorescence measurement

*P. pastoris* strains were pre-cultured in YPD medium to a log phase and then transferred to YNB medium containing the required carbon sources (1% glucose, 1% glycerol, 0.5% ethanol, 0.5% methanol) in a 24-well plate. Culture samples were collected at specific time points for fluorescence detection. GFP ﬂuorescence (normalized with OD_600nm_) from various samples was analyzed using a multi-mode microplate reader (Synergy 2, BioTek Instruments, USA).

### Production and activity assays of α-amylase

*P. pastoris* strains producing α-amylase were pre-cultured in YPD medium to a log phase and then transferred to BMY medium (2% tryptone, 1% yeast extract, 1.34% YNB, and 100 mmol/L potassium phosphate buffer, pH 6.0) supplemented with 1% methanol or 1% ethanol. Corresponding carbon sources were added every 24 h, and samples were collected to measure OD_600nm_ and enzyme activity. The enzyme activity of α-amylase was determined by the DNS method as previously reported (40).

### Statistical analysis

Data were obtained from three biological replicates from at least three experimental batches and presented as mean ± standard deviation. For transcriptomic analysis, gene expression was quantified using TPM. GraphPad Prism was used for Figure presentation and data analysis. The unpaired, two-tailed Student’s *t*-test was used to assess the differences among the groups. Statistical significance was set at *P* < 0.05 and *P* < 0.01.

## Data Availability

All data in the paper will be provided by authors upon request.

## SUPPLEMENTAL MATERIAL

Supplemental material aem.02191-24-s0001.docx Supplemental methods, Tables S1 to S4, and Figures S1 to S6.

ASM does not own the copyrights to Supplemental Material that may be linked to, or accessed through, an article. The authors have granted ASM a non-exclusive, world-wide license to publish the Supplemental Material files. Please contact the corresponding author directly for reuse.
