# Supplementary material for: Rational design and characterization of enhanced alcohol-inducible synthetic promoters in Pichia pastoris
Source: Appl Environ Microbiol. 2024 Dec 19;91(1):e02191-24. doi: 10.1128/aem.02191-24 (PMC11784102; doi:10.1128/aem.02191-24)
Supplement: Supplemental material — Supplemental methods, Tables S1 to S4, and Figures S1 to S6. [file aem.02191-24-s0001.docx]

Supplementary Material for

**Rational Design and Characterization of Enhanced Alcohol-Inducible Synthetic Promoters in *Pichia pastoris***

Qi Liu,^a^ Yun-hao Li,^a^ Liu-fei Tao,^a^ Jia-yi Yang,^a^ Yi-lun Zhang,^a^ Meng-hao Cai ^a,b^#

^a^State Key Laboratory of Bioreactor Engineering, East China University of Science and Technology, 130 Meilong Road, Shanghai 200237, China

^b^Shanghai Collaborative Innovation Center for Biomanufacturing, Shanghai 200237, China

#Address correspondence to Menghao Cai, cmh022199@ecust.edu.cn.

Qi Liu and Yunhao Li contributed equally to this work. Author order was determined by agreement of the authors.

**This supplementary file includes:**

Supplementary methods.

Tables S1 to S4

Figures S1 to S6

**Supplementary Methods**

Two fragments were amplified from a plasmid of pP-P*_AOX1_*G with primer pairs of pP-A1-F/GFP-AOX1-R and pP-A2-F/GFP-AOX1-R, and separately fused with a vector generated by digesting the plasmid pPlacO1cAG with *Sac*І/*Bam*HІ, resulting plasmids of pP-P*_A1_*G and pP-P*_A2_*G by gibson assembly. Two fragments were amplified from a plasmid of pP-P*_AOX1_*G with primer pairs of PA3-F/inOri-R and inOri-F/PA3-R, and then fused to produce a plasmid of pP-P*_A3_*G by gibson assembly. Similarly, the plasmids of pP-P*_A4_*G, pP-P*_A5_*G, pP-P*_A6_*G, pP-P*_A7_*G, pP-P*_A8_*G, pP-P*_A9_*G, pP-P*_A10_*G, and pP-P*_A11_*G were constructed. A fragment was amplified from the plasmid pP-P*_A3_*G with primer pair of pP-A2-F/GFP-AOX1-R, fused with a vector of *Sac*І/*Bam*HІ digested pPlacO1cAG to produce a plasmid of pP-P*_A12_*G. Subsequently, three fragments were amplified from a plasmid of pP-P*_A12_*G with primer pairs of PA12-F/inOri-R, inOri-F/PA12-R and PM-F/PM2-R, respectively. Then three fragments were fused to produce a plasmid of pP-P*_A13_*G. The plasmids of P*_A14_*G and P*_A15_*G were constructed by similar method.

The primer pair of pP-P0338-F/GFP-P0338-R was used to amplify the fragment containing P*_0338_* region, which was fused with a vector generated by digesting the plasmid pPlacO1cAG with *Sac*І/*Bam*HІ, resulting plasmids of pP-P*_0338_*G. Similarly, the plasmids harboring various ethanol-inducible promoters were constructed. For the plasmids containing synthetic promoters, the primer pair of AOX1-GFP-F/AOX1-GFP-R was used to amplify the vector fragment. The core promoter fragments were amplified from GS115 genome and fused with a vector fragment to produce the plasmids of Group I. Then the plasmids of Group II, Group III, and Group IV were constructed by similar method.

The α-amylase coding sequence was synthesized into plasmid pPIC9k with codon optimization by GenScript, resulting a plasmid of pP-P*_AOX1_*Amy. The fragment was amplified from the plasmid of pP-P*_AOX1_*Amy with primer pairs of 5AOX1/inOri-R, another fragment was amplified from the plasmid of pP-P*_A13_*G with primer pairs of inOri-F/5AOX1R. Two fragments were fused to produce a plasmid of pP-P*_A13_*Amy. Two fragments were amplified from pP-P*_0068_*G and pP-P*_synIV-5_*G with primer pairs of pP-P0688-F/αF-P0688-R and pP-P0074-F/αF-PDAS2-R, and separately fused with a vector generated by digesting the plasmid pP-P*_A13_*Amy with *Sac*І/*Bam*HІ, resulting plasmids of pP-P*_A1_*G and pP-P*_A2_*G

**Table S1 The information of 36 ethanol-responsive genes identified through transcriptome sequencing.**

| Gene symbol | TPM_E | TPM_D | Fold change (D/E) | Gene description |
| --- | --- | --- | --- | --- |
| PAS_chr1-4_0338 | 2284 | 56 | 0.024 | Isocitrate lyase, catalyzes the formation of succinate and glyoxylate from isocitrate |
| PAS_chr4_0688 | 6083 | 159 | 0.026 | Mitochondrial succinate-fumarate transporter |
| PAS_chr4_0043 | 5816 | 16 | 0.003 | Mitochondrial aldehyde dehydrogenase |
| PAS_chr4_0972 | 4157 | 164 | 0.039 | Hypothetical protein |
| PAS_chr4_0627 | 3591 | 430 | 0.120 | Plasma membrane localized protein that protects membranes from desiccation |
| PAS_chr2-1_0874 | 3458 | 403 | 0.117 | Hypothetical protein |
| PAS_chr2-2_0355 | 2791 | 129 | 0.046 | Mitochondrial inner membrane carnitine transporter |
| PAS_chr4_0733 | 1975 | 342 | 0.173 | Flavoprotein subunit of succinate dehydrogenase (Sdh1p, Sdh2p, Sdh3p, Sdh4p) |
| PAS_chr1-1_0118 | 1883 | 235 | 0.125 | Hypothetical protein |
| PAS_chr3_0408 | 1398 | 46 | 0.033 | Hypothetical protein |
| PAS_chr2-2_0206 | 1041 | 166 | 0.159 | Hypothetical protein |
| PAS_chr1-3_0104 | 3273 | 388 | 0.119 | Aconitase, required for the tricarboxylic acid (TCA) cycle and also independently required for mitoc |
| PAS_chr2-2_0127 | 2438 | 430 | 0.176 | Mitochondrial cytochrome-c peroxidase |
| PAS_chr1-4_0487 | 2347 | 359 | 0.153 | Putative protein of unknown function |
| PAS_chr1-1_0030 | 1913 | 165 | 0.086 | General amino acid permease |
| PAS_chr2-2_0283 | 1861 | 306 | 0.164 | Membrane anchor subunit of succinate dehydrogenase (Sdh1p, Sdh2p, Sdh3p, Sdh4p) |
| PAS_chr3_1111 | 1856 | 303 | 0.163 | Iron-sulfur protein subunit of succinate dehydrogenase (Sdh1p, Sdh2p, Sdh3p, Sdh4p) |
| PAS_chr2-2_0131 | 1331 | 69 | 0.052 | Catalase A, breaks down hydrogen peroxide in the peroxisomal matrix formed by acyl-CoA oxidase (Pox1) |
| PAS_chr3_0410 | 1096 | 191 | 0.174 | L-ornithine transaminase (OTAse) |
| PAS_chr1-1_0226 | 1048 | 155 | 0.148 | Vacuolar proteinase B (yscB), a serine protease of the subtilisin family |
| PAS_chr3_0440 | 934 | 14 | 0.015 | Lactate transporter |
| PAS_chr2-2_0288 | 910 | 16 | 0.017 | Arginase, responsible for arginine degradation |
| PAS_chr2-2_0267 | 843 | 29 | 0.034 | 3-ketoacyl-CoA thiolase with broad chain length specificity |
| PAS_chr3_0975 | 708 | 41 | 0.058 | Peroxisomal 2,4-dienoyl-CoA reductase, auxiliary enzyme of fatty acid beta-oxidation |
| PAS_chr1-4_0109 | 642 | 22 | 0.034 | Protein involved in iron metabolism in mitochondria |
| PAS_chr4_0828 | 555 | 6 | 0.011 | Myo-inositol transporter with strong similarity to the minor myo-inositol transporter Itr2p |
| PAS_chr2-2_0458 | 552 | 55 | 0.100 | Hypothetical protein |
| PAS_chr1-3_0017 | 928 | 28 | 0.031 | High-affinity cysteine-specific transporter with similarity to the Dal5p family of transporters |
| PAS_chr1-4_0074 | 756 | 27 | 0.036 | Outer mitochondrial carnitine acetyltransferase, minor ethanol-inducible enzyme |
| PAS_chr2-1_0143 | 751 | 107 | 0.142 | Conserved protein of the mitochondrial matrix, performs a scaffolding function during assembly of ir |
| PAS_chr1-3_0013 | 745 | 74 | 0.099 | Hypothetical protein |
| PAS_chr1-3_0016 | 658 | 25 | 0.038 | Multifunctional enzyme of the peroxisomal fatty acid beta-oxidation pathway |
| PAS_chr1-4_0538 | 608 | 16 | 0.026 | Fatty-acyl coenzyme A oxidase |
| PAS_chr1-1_0110 | 576 | 74 | 0.128 | Mitochondrial matrix protein involved in biogenesis of iron-sulfur (Fe/S) cluster of Fe/S proteins |
| PAS_chr1-1_0158 | 536 | 28 | 0.051 | Putative transmembrane protein involved in export of ammonia, a starvation signal |
| PAS_chr3_0937 | 512 | 73 | 0.142 | 3-hydroxyisobutyryl-CoA hydrolase, member of a family of enoyl-CoA hydratase/isomerases |

**Table S2** **Plasmids used in this study.**

| **Plasmids** | **Characteristics** | **Source** |
| --- | --- | --- |
| pP-P*_AOX1_*G | pPIC3.5k derivative containing P*_AOX1_*-GFP cassette | (23) |
| pPlacO1cAG | pPIC3.5k derivative containing *lacO1*-cP*_AOX1_*-GFP cassette | (23) |
| pP-P*_A1_*G | pPIC3.5k derivative containing P*_A1_*-GFP cassette | This study |
| pP-P*_A2_*G | pPIC3.5k derivative containing P*_A2_*-GFP cassette | This study |
| pP-P*_A3_*G | pPIC3.5k derivative containing P*_A3_*-GFP cassette | This study |
| pP-P*_A4_*G | pPIC3.5k derivative containing P*_A4_*-GFP cassette | This study |
| pP-P*_A5_*G | pPIC3.5k derivative containing P*_A5_*-GFP cassette | This study |
| pP-P*_A6_*G | pPIC3.5k derivative containing P*_A6_*-GFP cassette | This study |
| pP-P*_A7_*G | pPIC3.5k derivative containing P*_A7_*-GFP cassette | This study |
| pP-P*_A8_*G | pPIC3.5k derivative containing P*_A8_*-GFP cassette | This study |
| pP-P*_A9_*G | pPIC3.5k derivative containing P*_A9_*-GFP cassette | This study |
| pP-P*_A10_*G | pPIC3.5k derivative containing P*_A10_*-GFP cassette | This study |
| pP-P*_A11_*G | pPIC3.5k derivative containing P*_A11_*-GFP cassette | This study |
| pP-P*_A12_*G | pPIC3.5k derivative containing P*_A12_*-GFP cassette | This study |
| pP-P*_A13_*G | pPIC3.5k derivative containing P*_A13_*-GFP cassette | This study |
| pP-P*_A14_*G | pPIC3.5k derivative containing P*_A14_*-GFP cassette | This study |
| pP-P*_A15_*G | pPIC3.5k derivative containing P*_A15_*-GFP cassette | This study |
| pP-P*_0338_*G | pPIC3.5k derivative containing P*_0338_*-GFP cassette | This study |
| pP-P*_0688_*G | pPIC3.5k derivative containing P*_0688_*-GFP cassette | This study |
| pP-P*_0043_*G | pPIC3.5k derivative containing P*_0043_*-GFP cassette | This study |
| pP-P*_0972_*G | pPIC3.5k derivative containing P*_0972_*-GFP cassette | This study |
| pP-P*_0627_*G | pPIC3.5k derivative containing P*_0627_*-GFP cassette | This study |
| pP-P*_0874_*G | pPIC3.5k derivative containing P*_0874_*-GFP cassette | This study |
| pP-P*_0355_*G | pPIC3.5k derivative containing P*_0355_*-GFP cassette | This study |
| pP-P*_0733_*G | pPIC3.5k derivative containing P*_0733_*-GFP cassette | This study |
| pP-P*_0118_*G | pPIC3.5k derivative containing P*_0118_*-GFP cassette | This study |
| pP-P*_0408_*G | pPIC3.5k derivative containing P*_0408_*-GFP cassette | This study |
| pP-P*_0206_*G | pPIC3.5k derivative containing P*_0206_*-GFP cassette | This study |
| pP-P*_0104_*G | pPIC3.5k derivative containing P*_0104_*-GFP cassette | This study |
| pP-P*_0127_*G | pPIC3.5k derivative containing P*_0127_*-GFP cassette | This study |
| pP-P*_0487_*G | pPIC3.5k derivative containing P*_0487_*-GFP cassette | This study |
| pP-P*_0030_*G | pPIC3.5k derivative containing P*_0030_*-GFP cassette | This study |
| pP-P*_0283_*G | pPIC3.5k derivative containing P*_0283_*-GFP cassette | This study |
| pP-P*_1111_*G | pPIC3.5k derivative containing P*_1111_*-GFP cassette | This study |
| pP-P*_0131_*G | pPIC3.5k derivative containing P*_0131_*-GFP cassette | This study |
| pP-P*_0410_*G | pPIC3.5k derivative containing P*_0410_*-GFP cassette | This study |
| pP-P*_0226_*G | pPIC3.5k derivative containing P*_0226_*-GFP cassette | This study |
| pP-P*_0440_*G | pPIC3.5k derivative containing P*_0440_*-GFP cassette | This study |
| pP-P*_0288_*G | pPIC3.5k derivative containing P*_0288_*-GFP cassette | This study |
| pP-P*_0267_*G | pPIC3.5k derivative containing P*_0267_*-GFP cassette | This study |
| pP-P*_0975_*G | pPIC3.5k derivative containing P*_0975_*-GFP cassette | This study |
| pP-P*_0109_*G | pPIC3.5k derivative containing P*_0109_*-GFP cassette | This study |
| pP-P*_0828_*G | pPIC3.5k derivative containing P*_0828_*-GFP cassette | This study |
| pP-P*_0458_*G | pPIC3.5k derivative containing P*_0458_*-GFP cassette | This study |
| pP-P*_0017_*G | pPIC3.5k derivative containing P*_0017_*-GFP cassette | This study |
| pP-P*_0074_*G | pPIC3.5k derivative containing P*_0074_*-GFP cassette | This study |
| pP-P*_0143_*G | pPIC3.5k derivative containing P*_0143_*-GFP cassette | This study |
| pP-P*_0013_*G | pPIC3.5k derivative containing P*_0013_*-GFP cassette | This study |
| pP-P*_0016_*G | pPIC3.5k derivative containing P*_0016_*-GFP cassette | This study |
| pP-P*_0538_*G | pPIC3.5k derivative containing P*_0538_*-GFP cassette | This study |
| pP-P*_0110_*G | pPIC3.5k derivative containing P*_0110_*-GFP cassette | This study |
| pP-P*_0158_*G | pPIC3.5k derivative containing P*_0158_*-GFP cassette | This study |
| pP-P*_0937_*G | pPIC3.5k derivative containing P*_0937_*-GFP cassette | This study |
| pP-URS*_AOX1_*-cP*_GAP_*G | pPIC3.5k derivative containing URS*_AOX1_*-cP*_GAP_*-GFP cassette | This study |
| pP-URS*_AOX1_*-cP*_DAS1_*G | pPIC3.5k derivative containing URS*_AOX1_*-cP*_DAS1_*-GFP cassette | This study |
| pP-URS*_AOX1_*-cP*_DAS2_*G | pPIC3.5k derivative containing URS*_AOX1_*-cP*_DAS2_*-GFP cassette | This study |
| pP-URS*_AOX1_*-cP*_FLD1_*G | pPIC3.5k derivative containing URS*_AOX1_*-cP*_FLD1_*-GFP cassette | This study |
| pP-URS*_AOX1_*-cP*_ICL1_*G | pPIC3.5k derivative containing URS*_AOX1_*-cP*_ICL1_*-GFP cassette | This study |
| pP-URS*_AOX1_*-cP*_PHO89_*G | pPIC3.5k derivative containing URS*_AOX1_*-cP*_PHO89_*-GFP cassette | This study |
| pP-URS*_AOX1_*-cP*_THI11_*G | pPIC3.5k derivative containing URS*_AOX1_*-cP*_THI11_*-GFP cassette | This study |
| pP-URS*_AOX1_*-cP*_LRA3_*G | pPIC3.5k derivative containing URS*_AOX1_*-cP*_LRA3_*-GFP cassette | This study |
| pP-URS*_AOX1_*-cP*_TEF1_*G | pPIC3.5k derivative containing URS*_AOX1_*-cP*_TEF1_*-GFP cassette | This study |
| pP-URS*_AOX1_*-cP*_ENO1_*G | pPIC3.5k derivative containing URS*_AOX1_*-cP*_ENO1_*-GFP cassette | This study |
| pP-URS*_AOX1_*-cP*_GCW14_*G | pPIC3.5k derivative containing URS*_AOX1_*-cP*_GCW14_*-GFP cassette | This study |
| pP-URS*_AOX1_*-cP*_PET9_*G | pPIC3.5k derivative containing URS*_AOX1_*-cP*_PET9_*-GFP cassette | This study |
| pP-P*_syn1_*G | pPIC3.5k derivative containing URS*_A13_*-cP*_THI11_*-GFP cassette | This study |
| pP-P*_syn2_*G | pPIC3.5k derivative containing URS*_A13_*-cP*_GCW14_*-GFP cassette | This study |
| pP-P*_syn3_*G | pPIC3.5k derivative containing URS*_A13_*-cP*_GAP_*-GFP cassette | This study |
| pP-P*_syn4_*G | pPIC3.5k derivative containing URS*_A13_*-cP*_DAS2_*-GFP cassette | This study |
| pP-P*_syn5_*G | pPIC3.5k derivative containing URS*_A13_*-cP*_0074_*-GFP cassette | This study |
| pP-P*_syn6_*G | pPIC3.5k derivative containing URS*_0074_*-cP*_AOX1_*-GFP cassette | This study |
| pP-P*_syn7_*G | pPIC3.5k derivative containing URS*_0074_*-cP*_THI11_*-GFP cassette | This study |
| pP-P*_syn8_*G | pPIC3.5k derivative containing URS*_0074_*-cP*_GCW14_*-GFP cassette | This study |
| pP-P*_syn9_*G | pPIC3.5k derivative containing URS*_0074_*-cP*_GAP_*-GFP cassette | This study |
| pP-P*_syn10_*G | pPIC3.5k derivative containing URS*_0074_*-cP*_DAS2_*-GFP cassette | This study |
| pP-P*_AOX1_*Amy | pPIC3.5k derivative containing P*_AOX1_*-Amy cassette | This study |
| pP-P*_A13_*Amy | pPIC3.5k derivative containing URS*_A13_*-P*_A13_*-Amy cassette | This study |
| pP-P*_syn10_*Amy | pPIC3.5k derivative containing URS*_A13_*-P*_syn10_*-Amy cassette | This study |

**Table S3** **Strains used in this study.**

| **Strain** | **Genotype** | | **Source** |  |
| --- | --- | --- | --- | --- |
| ***Escherichia coli*** | | |  | |
| Top 10 | | F^-^[*lacI*^q^ Tn*10* (Tet^r^)] *mcrA* Φ80*lacZ* ΔM15 Δ*lac X74* *deoR* *recA1* | Invitrogen | |
| ***Pichia pastoris*** | | |  | |
| GS115 | | *his4* | Invitrogen | |
| GS_P*_AOX1_*-G | | GS115 *his4*::pP-P*_AOX1_*G (P*_AOX1_*-GFP *HIS4, KAN*) | (23) | |
| GS_P*_A2_*G | | GS115 *his4*::pP-P*_A2_*G, *HIS4* | This study | |
| GS_P*_A3_*G | | GS115 *his4*::pP-P*_A3_*G, *HIS4* | This study | |
| GS_P*_A4_*G | | GS115 *his4*::pP-P*_A4_*G, *HIS4* | This study | |
| GS_P*_A5_*G | | GS115 *his4*::pP-P*_A5_*G, *HIS4* | This study | |
| GS_P*_A6_*G | | GS115 *his4*::pP-P*_A6_*G, *HIS4* | This study | |
| GS_P*_A7_*G | | GS115 *his4*::pP-P*_A7_*G, *HIS4* | This study | |
| GS_P*_A8_*G | | GS115 *his4*::pP-P*_A8_*G, *HIS4* | This study | |
| GS_P*_A9_*G | | GS115 *his4*::pP-P*_A9_*G, *HIS4* | This study | |
| GS_P*_A10_*G | | GS115 *his4*::pP-P*_A10_*G, *HIS4* | This study | |
| GS_P*_A11_*G | | GS115 *his4*::pP-P*_A11_*G, *HIS4* | This study | |
| GS_P*_A12_*G | | GS115 *his4*::pP-P*_A12_*G, *HIS4* | This study | |
| GS_P*_A13_*G | | GS115 *his4*::pP-P*_A13_*G, *HIS4* | This study | |
| GS_P*_A14_*G | | GS115 *his4*::pP-P*_A14_*G, *HIS4* | This study | |
| GS_P*_A15_*G | | GS115 *his4*::pP-P*_A15_*G, *HIS4* | This study | |
| GS_P*_0338_*G | | GS115 *his4*::pP-P*_0338_*G, *HIS4* | This study | |
| GS_P*_0688_*G | | GS115 *his4*::pP-P*_0688_*G, *HIS4* | This study | |
| GS_P*_0043_*G | | GS115 *his4*::pP-P*_0043_*G, *HIS4* | This study | |
| GS_P*_0972_*G | | GS115 *his4*::pP-P*_0972_*G, *HIS4* | This study | |
| GS_P*_0627_*G | | GS115 *his4*::pP-P*_0627_*G, *HIS4* | This study | |
| GS_P*_0874_*G | | GS115 *his4*::pP-P*_0874_*G, *HIS4* | This study | |
| GS_P*_0355_*G | | GS115 *his4*::pP-P*_0355_*G, *HIS4* | This study | |
| GS_P*_0733_*G | | GS115 *his4*::pP-P*_0733_*G, *HIS4* | This study | |
| GS_P*_0118_*G | | GS115 *his4*::pP-P*_0118_*G, *HIS4* | This study | |
| GS_P*_0408_*G | | GS115 *his4*::pP-P*_0408_*G, *HIS4* | This study | |
| GS_P*_0206_*G | | GS115 *his4*::pP-P*_0206_*G, *HIS4* | This study | |
| GS_P*_0104_*G | | GS115 *his4*::pP-P*_0104_*G, *HIS4* | This study | |
| GS_P*_0127_*G | | GS115 *his4*::pP-P*_0127_*G, *HIS4* | This study | |
| GS_P*_0487_*G | | GS115 *his4*::pP-P*_0487_*G, *HIS4* | This study | |
| GS_P*_0030_*G | | GS115 *his4*::pP-P*_0030_*G, *HIS4* | This study | |
| GS_P*_0283_*G | | GS115 *his4*::pP-P*_0283_*G, *HIS4* | This study | |
| GS_P*_1111_*G | | GS115 *his4*::pP-P*_1111_*G, *HIS4* | This study | |
| GS_P*_0131_*G | | GS115 *his4*::pP-P*_0131_*G, *HIS4* | This study | |
| GS_P*_0410_*G | | GS115 *his4*::pP-P*_0410_*G, *HIS4* | This study | |
| GS_P*_0226_*G | | GS115 *his4*::pP-P*_0226_*G, *HIS4* | This study | |
| GS_P*_0440_*G | | GS115 *his4*::pP-P*_0440_*G, *HIS4* | This study | |
| GS_P*_0288_*G | | GS115 *his4*::pP-P*_0288_*G, *HIS4* | This study | |
| GS_P*_0267_*G | | GS115 *his4*::pP-P*_0267_*G, *HIS4* | This study | |
| GS_P*_0975_*G | | GS115 *his4*::pP-P*_0975_*G, *HIS4* | This study | |
| GS_P*_0109_*G | | GS115 *his4*::pP-P*_0109_*G, *HIS4* | This study | |
| GS_P*_0828_*G | | GS115 *his4*::pP-P*_0828_*G, *HIS4* | This study | |
| GS_P*_0458_*G | | GS115 *his4*::pP-P*_0458_*G, *HIS4* | This study | |
| GS_P*_0017_*G | | GS115 *his4*::pP-P*_0017_*G, *HIS4* | This study | |
| GS_P*_0074_*G | | GS115 *his4*::pP-P*_0074_*G, *HIS4* | This study | |
| GS_P*_0143_*G | | GS115 *his4*::pP-P*_0143_*G, *HIS4* | This study | |
| GS_P*_0013_*G | | GS115 *his4*::pP-P*_0013_*G, *HIS4* | This study | |
| GS_P*_0016_*G | | GS115 *his4*::pP-P*_0016_*G, *HIS4* | This study | |
| GS_P*_0538_*G | | GS115 *his4*::pP-P*_0538_*G, *HIS4* | This study | |
| GS_P*_0110_*G | | GS115 *his4*::pP-P*_0110_*G, *HIS4* | This study | |
| GS_P*_0158_*G | | GS115 *his4*::pP-P*_0158_*G, *HIS4* | This study | |
| GS_P*_0937_*G | | GS115 *his4*::pP-P*_0937_*G, *HIS4* | This study | |
| GS_URS*_AOX1_*-cP*_GAP_*G | | GS115 *his4*::pP-URS*_AOX1_*-cP*_GAP_*G, *HIS4* | This study | |
| GS_URS*_AOX1_*-cP*_DAS1_*G | | GS115 *his4*::pP-URS*_AOX1_*-cP*_DAS1_*G, *HIS4* | This study | |
| GS_URS*_AOX1_*-cP*_DAS2_*G | | GS115 *his4*::pP-URS*_AOX1_*-cP*_DAS2_*G, *HIS4* | This study | |
| GS_URS*_AOX1_*-cP*_FLD1_*G | | GS115 *his4*::pP-URS*_AOX1_*-cP*_FLD1_*G, *HIS4* | This study | |
| GS_URS*_AOX1_*-cP*_ICL1_*G | | GS115 *his4*::pP-URS*_AOX1_*-cP*_ICL1_*G, *HIS4* | This study | |
| GS_URS*_AOX1_*-cP*_PHO89_*G | | GS115 *his4*::pP-URS*_AOX1_*-cP*_PHO89_*G, *HIS4* | This study | |
| GS_URS*_AOX1_*-cP*_THI11_*G | | GS115 *his4*::pP-URS*_AOX1_*-cP*_THI11_*G, *HIS4* | This study | |
| GS_URS*_AOX1_*-cP*_LRA3_*G | | GS115 *his4*::pP-URS*_AOX1_*-cP*_LRA3_*G, *HIS4* | This study | |
| GS_URS*_AOX1_*-cP*_TEF1_*G | | GS115 *his4*::pP-URS*_AOX1_*-cP*_TEF1_*G, *HIS4* | This study | |
| GS_URS*_AOX1_*-cP*_ENO1_*G | | GS115 *his4*::pP-URS*_AOX1_*-cP*_ENO1_*G, *HIS4* | This study | |
| GS_URS*_AOX1_*-cP*_GCW14_*G | | GS115 *his4*::pP-URS*_AOX1_*-cP*_GCW14_*G, *HIS4* | This study | |
| GS_URS*_AOX1_*-cP*_PET9_*G | | GS115 *his4*::pP-URS*_AOX1_*-cP*_PET9_*G, *HIS4* | This study | |
| GS_P*_synII-1_*G | | GS115 *his4*::pP-URS*_A13_*-cP*_THI11_*G, *HIS4* | This study | |
| GS_P*_synII-2_*G | | GS115 *his4*::pP-URS*_A13_*-cP*_GCW14_*G, *HIS4* | This study | |
| GS_P*_synII-3_*G | | GS115 *his4*::pP-URS*_A13_*-cP*_GAP_*G, *HIS4* | This study | |
| GS_P*_synII-4_*G | | GS115 *his4*::pP-URS*_A13_*-cP*_DAS2_*G, *HIS4* | This study | |
| GS_P*_synIII-1_*G | | GS115 *his4*::pP-URS*_0688_*-cP*_AOX1_*G, *HIS4* | This study | |
| GS_P*_synIII-2_*G | | GS115 *his4*::pP-URS*_0688_*-cP*_THI11_*G, *HIS4* | This study | |
| GS_P*_synIII-3_*G | | GS115 *his4*::pP-URS*_0688_*-cP*_GCW14_*G, *HIS4* | This study | |
| GS_P*_synIII-4_*G | | GS115 *his4*::pP-URS*_0688_*-cP*_GAP_*G, *HIS4* | This study | |
| GS_P*_synIII-5_*G | | GS115 *his4*::pP-URS*_0688_*-cP*_DAS2_*G, *HIS4* | This study | |
| GS_P*_synIV-1_*G | | GS115 *his4*::pP-URS*_0074_*-cP*_AOX1_*G, *HIS4* | This study | |
| GS_P*_synIV-2_*G | | GS115 *his4*::pP-URS*_0074_*-cP*_THI11_*G, *HIS4* | This study | |
| GS_P*_synIV-3_*G | | GS115 *his4*::pP-URS*_0074_*-cP*_GCW14_*G, *HIS4* | This study | |
| GS_P*_synIV-4_*G | | GS115 *his4*::pP-URS*_0074_*-cP*_GAP_*G, *HIS4* | This study | |
| GS_P*_synIV-5_*G | | GS115 *his4*::pP-URS*_0074_*-cP*_DAS2_*G, *HIS4* | This study | |
| GS_P*_AOX1_*Amy | | GS115 *his4*::pP-P*_AOX1_*Amy, *HIS4* | This study | |
| GS_P*_A13_*Amy | | GS115 *his4*::pP-P*_A13_*Amy, *HIS4* | This study | |
| GS_P*_0688_*Amy | | GS115 *his4*::pP-P*_0688_*Amy, *HIS4* | This study | |
| GS_P*_synIV-5_*Amy | | GS115 *his4*::pP-P*_synIV-5_*Amy, *HIS4* | This study | |

**Table S4** **Primers used in this study.**

| **Primer** | **Sequence (5’-3’)** |
| --- | --- |
| pP-A1-F | ATAGACGCAGATCGGGAACGAGCTGGAGGGGATACACTAGCAGC |
| pP-A2-F | ATAGACGCAGATCGGGAACGAGCTCTTCTATTAGGCTACTAACACC |
| GFP-AOX1-R | CTTTAGAACCCATGGTGTAGGATCCGTTTCGAATAATTAGTTGT |
| inOri-F | TACCTGTCCGCCTTTCTCCC |
| inOri-R | GGGAGAAAGGCGGACAGGTA |
| PA3-F | ACTTTATTAGCCTGTCTATCCTCCGCATTACACCCGAAC |
| PA3-R | GATAGACAGGCTAATAAAGTCATGG |
| PA4-F | GGCTTTCTGAGTGTGGGGTCGTCTTGGAACCTAATATGACAAAAGCG |
| PA4-R | GACCCCACACTCAGAAAGCC |
| PA5-F | ACTTTATTAGCCTGTCTATCGTCTTGGAACCTAATATGAC |
| PcA-F | TAACCCCTACTTGACAGCAA |
| PA6-F | CTGTCTTGGAACCTAATATGGCTAACGGCCAGTTGGTC |
| PA6-R | CATATTAGGTTCCAAGACAGCG |
| PA7-F | TTGTCTCCACATTGTATGCTTC |
| PA7-R | AGCATACAATGTGGAGACAAACAGGTGCACCGGGGTTCAG |
| PA8-F | TGATTATGCATTGTCTCCACTAACGTTCATGATCAAAATTTAACTG |
| PA8-R | GTGGAGACAATGCATAATCATC |
| PA9-F | GGAATACTGCTGATAGCCTATATATAAACAGAAGGAAGCTGCC |
| PA9-R | TAGGCTATCAGCAGTATTCC |
| PA10-R | TTGCTGTCAAGTAGGGGTTAGTGGAGACAATGCATAATCATC |
| PA11-R | TTGCTGTCAAGTAGGGGTTAACAGGTGCACCGGGGTTCAG |
| PA12-F | CTTCTATTAGGCTACTAACACC |
| PA12-R | AGCTCGTTCCCGATCTGCGTC |
| PM-F | GACGCAGATCGGGAACGAGCTCTTCTATTAGGCTACTAACACC |
| PM2-R | TGTTAGTAGCCTAATAGAAGGATAGACAGGCTAATAAAGTCATG |
| PM4-R | TGTTAGTAGCCTAATAGAAGCCCCACACTCAGAAAGCCC |
| PM7-R | TGTTAGTAGCCTAATAGAAGGTTAGCATTTCAACGAACCAAAC |
| pP-P0013-F | ATAGACGCAGATCGGGAACGAGCTGATTCGGGTCTACAATATACTGTTCC |
| GFP-P0013-R | CTTTAGAACCCATGGTGTAGGATCTGAGGAGTAAAGGTGAATATATTGATAG |
| pP-P0016-F | ATAGACGCAGATCGGGAACGAGCTCTGGTCTTCAGCCATATTGG |
| GFP-P0016-R | CTTTAGAACCCATGGTGTAGGATCTTTAGTACCAACTACAACTTCAATTC |
| pP-P0017-F | ATAGACGCAGATCGGGAACGAGCTACCAAAGCATCGATTAACTCTC |
| GFP-P0017-R | CTTTAGAACCCATGGTGTAGGATCTTTGGTTTCTAACACGATTATTCAG |
| pP-P0030-F | ATAGACGCAGATCGGGAACGAGCTGAGCAGCTTCTCATTGAGGT |
| GFP-P0030-R | CTTTAGAACCCATGGTGTAGGATCTATAAGATAGTTGTGTTGACGG |
| pP-P0074-F | ATAGACGCAGATCGGGAACGAGCTTGAATTCCTGGCTGCCCACA |
| GFP-P0074-R | CTTTAGAACCCATGGTGTAGGATCGGTGCCAAACAGAAGTGTAA |
| pP-P0104-F | ATAGACGCAGATCGGGAACGAGCTTTCATCAGTTGCCCATTCAC |
| GFP-P0104-R | CTTTAGAACCCATGGTGTAGGATCACTGTATAACGGGAACAAAAGGG |
| pP-P0109-F | ATAGACGCAGATCGGGAACGAGCTAGATATGTCCTTCCTAACCA |
| GFP-P0109-R | CTTTAGAACCCATGGTGTAGGATCCTTGCAAATATGATCTGTATTTGAG |
| pP-P0110-F | ATAGACGCAGATCGGGAACGAGCTAAAGGTGAGTTAGTGGATGA |
| GFP-P0110-R | CTTTAGAACCCATGGTGTAGGATCGATTTCTTATTGTACAGCAAAAAAATGAGG |
| pP-P0118-F | ATAGACGCAGATCGGGAACGAGCTTCAATTTGGAAAACTGGCGC |
| GFP-P0118-R | CTTTAGAACCCATGGTGTAGGATCTTCTCCTATAAAAATACCTATGGGAAC |
| pP-P0127-F | ATAGACGCAGATCGGGAACGAGCTCATTCGGAGACTGTGATCTCTC |
| GFP-P0127-R | CTTTAGAACCCATGGTGTAGGATCGTTAAAAAGTATAATAGCCTAGGAAG |
| pP-P0131-F | ATAGACGCAGATCGGGAACGAGCTCTATGTTGACGGAGAGTGTTG |
| GFP-P0131-R | CTTTAGAACCCATGGTGTAGGATCTTTAATTGTAAGTCTTGACTAGAGC |
| pP-P0143-F | ATAGACGCAGATCGGGAACGAGCTATTGTTTTCATAATGGTTACGG |
| GFP-P0143-R | CTTTAGAACCCATGGTGTAGGATCGTTTAAAGCTATTCGGTTATTG |
| pP-P0158-F | ATAGACGCAGATCGGGAACGAGCTTGTTGCTGCTTAGTAAATTAG |
| GFP-P0158-R | CTTTAGAACCCATGGTGTAGGATCAGTTTAGTTTATAATTAAGAAGGTGTGTAG |
| pP-P0206-F | ATAGACGCAGATCGGGAACGAGCTATAGCCCATAATACTTCCTAAC |
| GFP-P0206-R | CTTTAGAACCCATGGTGTAGGATCTATTGAACAATAGAGTTATATTGGTGG |
| pP-P0226-F | ATAGACGCAGATCGGGAACGAGCTAGTTTAAATAATATGGAGATTTGTTTGAAGAG |
| GFP-P0226-R | CTTTAGAACCCATGGTGTAGGATCAATAACTTCATGACTGCATTTG |
| pP-P0267-F | ATAGACGCAGATCGGGAACGAGCTTCAGGATGATTTCATAAGTGTC |
| GFP-P0267-R | CTTTAGAACCCATGGTGTAGGATCCCCTGTTTACTATAACTGAAAAG |
| pP-P0283-F | ATAGACGCAGATCGGGAACGAGCTTTCCCTGGTTTCACGTTTTG |
| GFP-P0283-R | CTTTAGAACCCATGGTGTAGGATCTGGTGTTAGTAGGGATACCG |
| pP-P0288-F | ATAGACGCAGATCGGGAACGAGCTCCAAACTTATGGAAGAACTTG |
| GFP-P0288-R | CTTTAGAACCCATGGTGTAGGATCGATGAGTTACAGAGAGTACG |
| pP-P0355-F | ATAGACGCAGATCGGGAACGAGCTTCTCCATCTCTTTCAATTGC |
| GFP-P0355-R | CTTTAGAACCCATGGTGTAGGATCCGTTCTAGAAATTCACAGGC |
| pP-P0408-F | ATAGACGCAGATCGGGAACGAGCTATGAAAGCGCGAAAGAGAG |
| GFP-P0408-R | CTTTAGAACCCATGGTGTAGGATCCTTTGAGAATAGATTAGAGCAG |
| pP-P0410-F | ATAGACGCAGATCGGGAACGAGCTAGAGCCATGAGTTTGTGATG |
| GFP-P0410-R | CTTTAGAACCCATGGTGTAGGATCTGAATATAAAAATATCAACTAGTTCTAGTTTG |
| pP-P0440-F | ATAGACGCAGATCGGGAACGAGCTATCTATCCTTTTACAAACAACAACAAC |
| GFP-P0440-R | CTTTAGAACCCATGGTGTAGGATCGGCGTTAGACTCTGTCTAGAG |
| pP-P0458-F | ATAGACGCAGATCGGGAACGAGCTGGAGGAGCAGGTCAAATCG |
| GFP-P0458-R | CTTTAGAACCCATGGTGTAGGATCTAGGGAGGTGAGTTGTAATC |
| pP-P0487-F | ATAGACGCAGATCGGGAACGAGCTACTAAATCTTCTCTCCCAAATGG |
| GFP-P0487-R | CTTTAGAACCCATGGTGTAGGATCGGTTTGGTTTGTGTATAGTTTGATGG |
| pP-P0538-F | ATAGACGCAGATCGGGAACGAGCTATAGCATAGAACAAGAATGGAAGC |
| GFP-P0538-R | CTTTAGAACCCATGGTGTAGGATCGTTGTGATTTTCAGTTGTTAGACTGG |
| pP-P0627-F | ATAGACGCAGATCGGGAACGAGCTGATAAAGAACGACCGGAACC |
| GFP-P0627-R | CTTTAGAACCCATGGTGTAGGATCTTCTAGTTGTGTTTGTGTTTTG |
| pP-P0688-F | ATAGACGCAGATCGGGAACGAGCTTTTTTTTTTTCTTTGGCGCTCTATGCG |
| GFP-P0688-R | CTTTAGAACCCATGGTGTAGGATCTTTGGAGAAGTTGGATATGGGG |
| pP-P0733-F | ATAGACGCAGATCGGGAACGAGCTTTCTTAGGCTCATCAGTTTATCC |
| GFP-P0733-R | CTTTAGAACCCATGGTGTAGGATCGGGGTAGATGATGAAGATGG |
| pP-P0828-F | ATAGACGCAGATCGGGAACGAGCTAAAGAGATACAAGCTAAACAAGACC |
| GFP-P0828-R | CTTTAGAACCCATGGTGTAGGATCGTCTATAAAACTTTAATAGAGAGGG |
| pP-P0874-F | ATAGACGCAGATCGGGAACGAGCTTTTTTGTTTTTAGAGTAATAAGGGTAGC |
| GFP-P0874-R | CTTTAGAACCCATGGTGTAGGATCGATATCGGGTACAACTAGATTCG |
| pP-P0937-F | ATAGACGCAGATCGGGAACGAGCTTGGTTGGAATGAAGCGCTAT |
| GFP-P0937-R | CTTTAGAACCCATGGTGTAGGATCACTGGTTCGGGGGTTGGAGC |
| pP-P0972-F | ATAGACGCAGATCGGGAACGAGCTGTGAACGATGGCGTATATTT |
| GFP-P0972-R | CTTTAGAACCCATGGTGTAGGATCTATATTAGAACGGTAGGAAGTT |
| pP-P0975-F | ATAGACGCAGATCGGGAACGAGCTAGCAAGTGCTCTAGACAGAG |
| GFP-P0975-R | CTTTAGAACCCATGGTGTAGGATCTCTTTGGTGAGTTAGAGAATCG |
| pP-P1111-F | ATAGACGCAGATCGGGAACGAGCTCCGGACTTTTAGTCTCATGT |
| GFP-P1111-R | CTTTAGAACCCATGGTGTAGGATCTGTATTAAGGATTATGCTGTTTATTG |
| pP-P0043-F | ATAGACGCAGATCGGGAACGAGCTTGGACTGTTCAATTTGAAGTCG |
| GFP-P0043-R | CTTTAGAACCCATGGTGTAGGATCGGATAAAGGTAAGGGAAAAAAGCA |
| pP-P0338-F | ATAGACGCAGATCGGGAACGAGCTTAACACTTTGTATAGCACATCGTAC |
| GFP-P0338-R | CTTTAGAACCCATGGTGTAGGATCTCTTGATATACTTGATACTGTGTTCTTTG |
| AOX1-cENO1-F | TAACCCCTACTTGACAGCAACCACCCTGGTCCTGAAAAG |
| AOX1-cENO1-R | TAGAACCCATGGTGTAGGATTTTTAGATGTAGATTGTTAT |
| AOX1-cDAS1-F | TAACCCCTACTTGACAGCAAAACTAAAGCAGGATGCCTG |
| AOX1-cDAS1-R | TAGAACCCATGGTGTAGGATTTTGTTCGATTATTCTCCAG |
| AOX1-cLRA3-F | TAACCCCTACTTGACAGCAATGGGGTTGAAAGATGGAGAC |
| AOX1-cLRA3-R | TAGAACCCATGGTGTAGGATATTTTTAGGAGATAAAAATTC |
| AOX1-cICL1-F | TAACCCCTACTTGACAGCAACGAGAGTCCGCTACCCCAG |
| AOX1-cICL1-R | TAGAACCCATGGTGTAGGATTCTTGATATACTTGATACTGTG |
| AOX1-cPET9-F | TAACCCCTACTTGACAGCAATTTTTTTTTCCCAAAATCGC |
| AOX1-cPET9-R | TAGAACCCATGGTGTAGGATTTTGGAATATTATAGATTTG |
| AOX1-cTHI11-F | TAACCCCTACTTGACAGCAAGGAATGTTGGTAGCAATTGG |
| AOX1-cTHI11-R | TAGAACCCATGGTGTAGGATGATGATTTATTGAAGTTTCC |
| AOX1-cTEF1-F | TAACCCCTACTTGACAGCAATACACAAGATTTTCAGCAG |
| AOX1-cTEF1-R | TAGAACCCATGGTGTAGGATGTTGGCGAATAACTAAAATG |
| AOX1-cPHO89-F | TAACCCCTACTTGACAGCAAAAAGTGAAGCCGACTTTAGG |
| AOX1-cPHO89-R | TAGAACCCATGGTGTAGGATTGTGAATGATTATAAGATGAG |
| AOX1-cDAS2-F | TAACCCCTACTTGACAGCAATTAAGGGTTAACCGCCAAAT |
| AOX1-cDAS2-R | TAGAACCCATGGTGTAGGATTTTTGATGTTTGATAGTTTGATAAGAGTG |
| AOX1-cGAP-F | TAACCCCTACTTGACAGCAATGGAAACCACCAGAATCGAA |
| AOX1-cGAP-R | TAGAACCCATGGTGTAGGATTGTGTTTTGATAGTTGTTCAATTG |
| AOX1-cFLD1-F | TAACCCCTACTTGACAGCAAAATCAGGATTTCACTACTCA |
| AOX1-cFLD1-R | TAGAACCCATGGTGTAGGATTGTGAATATCAAGAATTGTATG |
| AOX1-cGCW14-F | TAACCCCTACTTGACAGCAATTTCGCCTGGTGCCGTACGG |
| AOX1-cGCW14-R | TAGAACCCATGGTGTAGGATTTTGTTGTTGAGTGAAGCGAG |
| AOX1-GFP-F | ATCCTACACCATGGGTTCTAAAGG |
| AOX1-GFP-R | TTGCTGTCAAGTAGGGGTTAG |
| cP-P0688-R | GGGGAACGATCCCAGTTGAT |
| P0688-cAOX1-F | ATCAACTGGGATCGTTCCCCTAACCCCTACTTGACAGCAA |
| P0688-cDAS2-F | ATCAACTGGGATCGTTCCCCTTAAGGGTTAACCGCCAAAT |
| P0688-cGAP-F | ATCAACTGGGATCGTTCCCCTGGAAACCACCAGAATCGAA |
| P0688-cGCW14-F | ATCAACTGGGATCGTTCCCCTTTCGCCTGGTGCCGTACGG |
| P0688-cTHI11-F | ATCAACTGGGATCGTTCCCCGGAATGTTGGTAGCAATTGG |
| cP-P0074-R | GAGCTGTGATGGGATGCCTT |
| P0074-cAOX1-F | AAGGCATCCCATCACAGCTCTAACCCCTACTTGACAGCAA |
| P0074-cDAS2-F | AAGGCATCCCATCACAGCTCTTAAGGGTTAACCGCCAAAT |
| P0074-cGAP-F | AAGGCATCCCATCACAGCTCTGGAAACCACCAGAATCGAA |
| P0074-cGCW14-F | AAGGCATCCCATCACAGCTCTTTCGCCTGGTGCCGTACGG |
| P0074-cTHI11-F | AAGGCATCCCATCACAGCTCGGAATGTTGGTAGCAATTGG |
| 5AOX1 | GACTGGTTCCAATTGACAAGC |
| 5AOX1R | GCTTGTCAATTGGAACCAGTC |
| αF-P0688-R | GAAGGAAATCTCATCGTTTGGATCTTTGGAGAAGTTGGATATGGG |
| αF-PDAS2-R | GATCCAAACGATGAGATTTCCTTCTTTTGATGTTTGATAGTTTGATAAGAGTG |
| pAOX1UP F | TCTCATGTTTGACAGCTTATCATC |
| yz-GFP-R | CTGGTCTTGTAGTTACCGTC |
| 3AOX1 | GCAAATGGCATTCTGACATCC |
| CN-gHisDO R | TCCCCAATCACTTGAGTACG |
| CN-gHisUP F | CCTACGAACTTGAGTATGGC |
| CN-pHisDO R | TGGTATGAGTCAGCAACACC |
| CN-pHisUP F | TGCTAGCGCTATATGCGTTG |

**
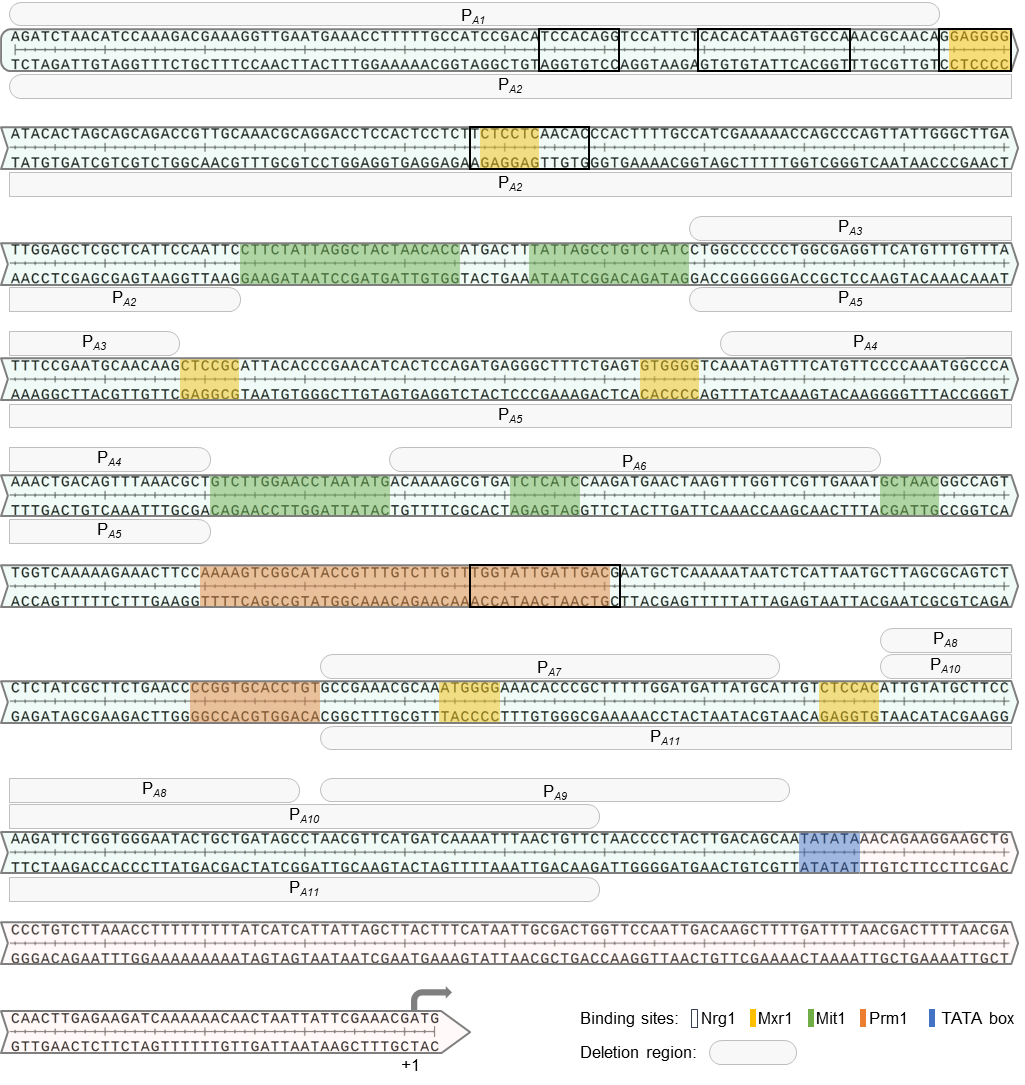
**

Fig**.** S1 Design schematic of the P*_AOX1_* mutants. The 940 bp sequence preceding the start codon (ATG) is shown as the P*_AOX1_* sequence. The binding sites for key transcription factors (Nrg1, Mxr1, Mit1, Prm1) and the TATA box are highlighted in different colors. The gray bars represent the deleted region of corresponding P*_AOX1_* mutants.

**
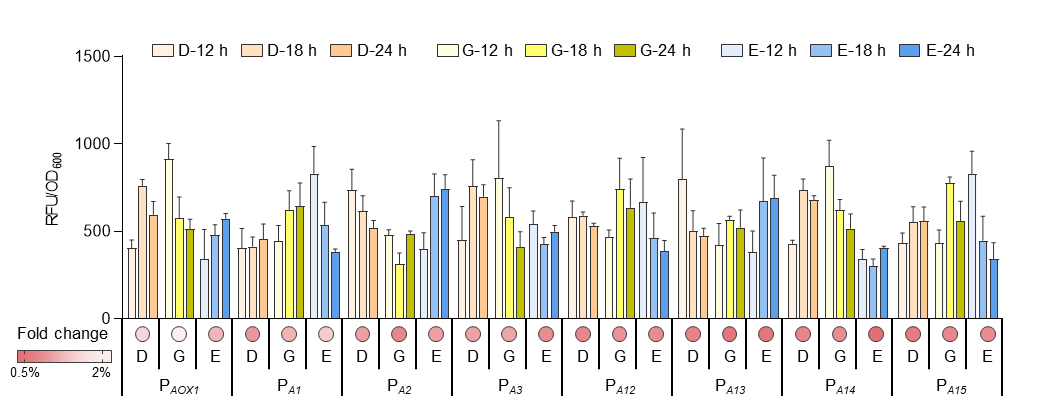
**

Fig**.** S2 Leakage expression of P*_AOX1_* mutants in various carbon source. The expression level of P*_AOX1_* mutants was measured in glucose (D), glycerol (G), ethanol (E) conditions. The circles represent the fold change, which is the ratio of the expression level of each P*_AOX1_* mutant under the corresponding carbon source to the that observed under methanol conditions (Fig. 1).

**
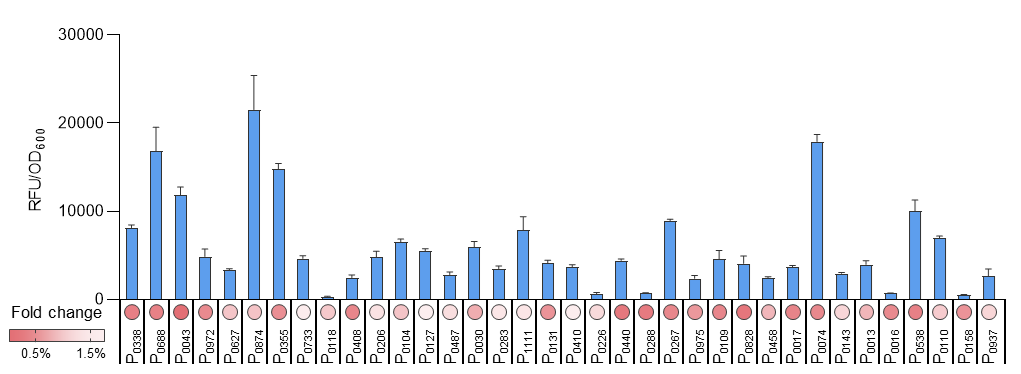
**

Fig**.** S3 Screening of ethanol-inducible promoters by RNA-seq and fluorescence detection. The 36 ethanol-inducible promoters were screened through RNA-seq analysis of the GS115 strain under ethanol and glucose conditions, respectively. The circles represent the fold change, which is the ratio of the transcription levels of each gene under the glucose compared to that under ethanol conditions. The GFP expression levels of selected promoters were measured after 24 hours in ethanol conditions.

**
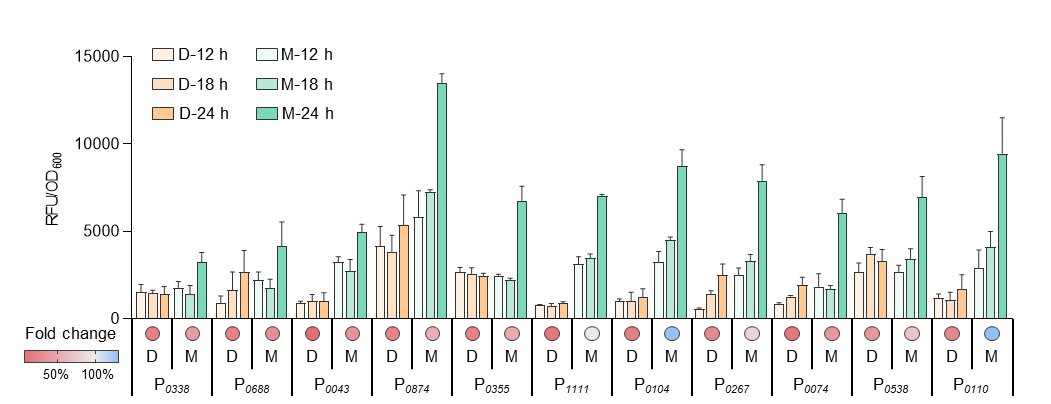
**

Fig**.** S4 Leakage expression of ethanol-inducible promoters in glucose and methanol. The expression level of 11 selected ethanol-inducible promoters were measured in glucose (D) and methanol (M) conditions. The circles represent the fold change, which is the ratio of the expression level of each promoter under the corresponding carbon source to the that observed under ethanol conditions (Fig. 1).

**
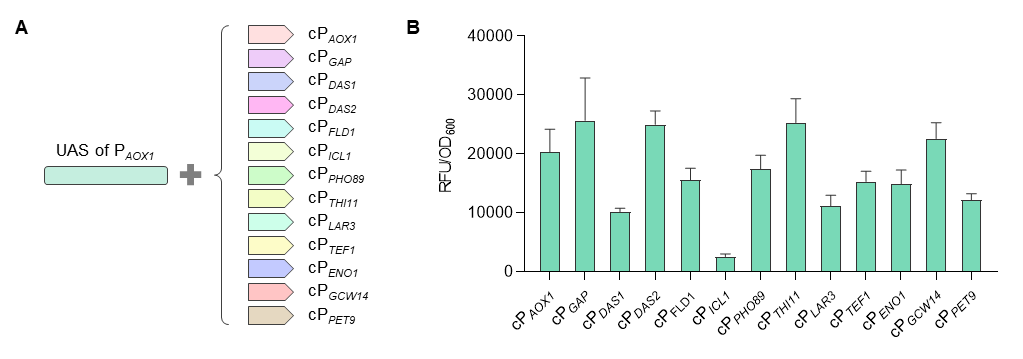
**

Fig**.** S5 Screening of core promotors by fusion with URS of P*_AOX1_*. (A) The 13 endogenous core promoters from *P. pastoris* were selected for fusion with the URS of the P*_AOX1_* to identify highly active core promoters. (B) The expression levels of the synthetic promoters were measured after 24 h under methanol conditions.


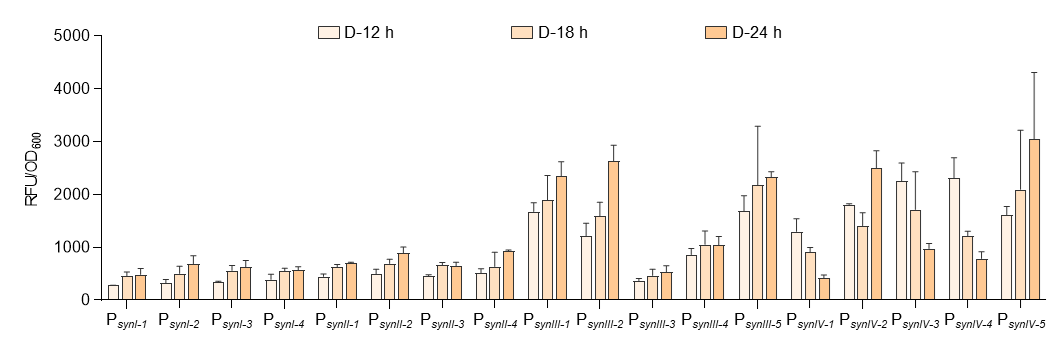


Fig**.** S6 Leakage expression of four group of synthetic promoters in glucose (D).
